# Retraction: Color tuning of Bi^3+^-doped double-perovskite Ba_2_(Gd_1−*x*_,Lu_*x*_)NbO_6_ (0 ≤ *x* ≤ 0.6) solid solution compounds *via* crystal field modulation for white LEDs

**DOI:** 10.1039/d4ra90157f

**Published:** 2025-01-10

**Authors:** Xufang Tang, Dingfeng Jin, Jun Zhao, Min Jin

**Affiliations:** a College of Materials and Chemistry, China Jiliang University Hangzhou 310018 P. R. China dfjin@cjlu.edu.cn; b Sichuan College of Architectural Technology Deyang 618000 P. R. China; c Department of Physics, Sichuan University Huanlu Nan No. 21 Chengdu 610041 Sichuan P. R. China superman_minjin@163.com

## Abstract

Retraction of ‘Color tuning of Bi^3+^-doped double-perovskite Ba_2_(Gd_1−*x*_,Lu_*x*_)NbO_6_ (0 ≤ *x* ≤ 0.6) solid solution compounds *via* crystal field modulation for white LEDs’ by Xufang Tang *et al.*, *RSC Adv.*, 2020, **10**, 25500–25508, https://doi.org/10.1039/D0RA03793A.

The Royal Society of Chemistry hereby wholly retracts this *RSC Advances* article due to evidence of systematic manipulation of the publication process affecting this article.

Ref. 28 and 33–39 are irrelevant and inappropriate. These references have been used in several other papers by the authors.^1^

The authors were contacted but did not provide a response to the concerns.

Given the significance of these concerns, the Editor has lost confidence in the authenticity of the findings presented in this paper.

The authors did not respond to any correspondence regarding the retraction of this article.

Signed: Laura Fisher, Executive Editor, *RSC Advances*

Date: 4th December 2024
